# The impact of ethnic minority status on tuberculosis diagnosis and treatment delays in Hunan Province, China

**DOI:** 10.1186/s12879-022-07072-4

**Published:** 2022-01-26

**Authors:** Beth Gilmour, Zuhui Xu, Liqiong Bai, Kefyalew Addis Alene, Archie C. A. Clements

**Affiliations:** 1 Faculty of Health Sciences, Curtin University, Western Australia, Kent St, Bentley, WA 6102 Australia; 2 Telethon Kids Institute, Nedlands, WA Australia; 3 Xiangya School of Public Health, Central South University, Changsha, China; 4 TB Control Institute of Hunan Province, Changsha, China

**Keywords:** Tuberculosis, Ethnic minority, Diagnosis delay, Treatment delay, China

## Abstract

**Background:**

Tuberculosis (TB) continues to be a major public health challenge in China. Understanding TB management delays within the context of China’s unique ethnic diversity may be of value in tackling the disease. This study sought to evaluate the impact of ethnic minority status on TB diagnosis and treatment delays.

**Methods:**

This retrospective cohort study was conducted on patients diagnosed with TB in Hunan Province, China between 2013 and 2018. Diagnosis delay was defined as the time interval between the onset of symptoms and the date of diagnosis. Treatment delay was defined as the time interval between diagnosis and treatment commencement. Univariable and multivariable logistic regression models were used to identify factors associated with TB diagnosis and treatment delay, including ethnic minority status. Adjusted odds ratios (AOR) with 95% confidence intervals (CI) were calculated to assess the strength of association between the dependant and independent variables.

**Results:**

A total of 318,792 TB patients were included in the study with a mean age of 51.7 years (SD 17.7). The majority of patients were male (72.6%) and Han ethnicity (90.6%). The odds of experiencing diagnosis delay (> 21 days) were significantly higher for Tujia (AOR: 1.46, 95% CI: 1.41, 1.51), Miao (AOR: 1.31, 95% CI: 1.26, 1.37), Dong (AOR: 1.97, 95% CI: 1.85, 2.11), Yao (AOR: 1.27, 95% CI: 1.17, 1.37), and Bai (AOR: 1.45, 95% CI: 1.22, 1.74) ethnic minorities compared to the Han majority. The odds of experiencing treatment delay (> 15 days) were significantly lower for five of the seven ethnic minority groups relative to the Han majority: Tujia (AOR 0.92, 95% CI 0.88, 0.96), Miao (AOR 0.74, 95% CI 0.70, 0.79), Dong (AOR 0.87, 95% CI 0.81, 0.95), Yao (AOR 0.20, 95% CI 0.17, 0.24) and ‘other’ (ethnic minorities that individually represented < 0.1% of the patient population) (AOR 0.70, 955 CI 0.51, 0.97).

**Conclusions:**

This study shows ethnic minority status to be a significant risk factor in diagnosis delay, but for it to reduce the odds of treatment delay. Further research is required to determine the underlying causes of diagnosis delay within ethnic minority populations.

**Supplementary Information:**

The online version contains supplementary material available at 10.1186/s12879-022-07072-4.

## Background

Tuberculosis (TB) is currently second to Coronavirus Disease 2019 (COVID -19) as a leading cause of death from a single infectious agent [1], claiming a life every 22 s [2]. Prior to the COVID-19 pandemic, TB was the leading cause of death [3] and throughout history is thought to have claimed more lives than any other microorganism [4]. TB is caused by *Mycobacterium tuberculosis* (MTB), an airborne pathogen that most commonly affects the lungs (pulmonary TB) albeit the pathogen can affect all organs (extrapulmonary TB). Although the disease can be cured, escalating drug resistance presents a global health security threat [3].

In 2019, China ranked third for the greatest number of new TB cases globally [3] with 833,000 people falling ill to the disease [5]. To address the burden of disease, China is in the process of comprehensive public health system reforms, including setting the goal of universal health coverage and transformation of the TB service delivery model [6–8]. In 1991, China launched its National Tuberculosis Control Programme (NTP) based on the World Health Organization (WHO) recommended Directly Observed Treatment Short-course (DOTS) strategy. The NTP aims to provide TB diagnosis and treatment services free of charge, with a focus on the poor, ethnic minorities and other vulnerable population groups. [9, 10].

Fundamental to the success of national TB control programs, is early detection and prompt and appropriate treatment [11]. Delays in timely diagnosis and treatment lead to disease progression, poor treatment outcomes, increased risk of transmission and an exacerbation of the socioeconomic consequences of the disease. [12].

A systematic review and meta-analysis of patient and diagnosis delays in China, found an array of contributing factors [13]. Factors included indicators of low socio-economic status (e.g., low level of education, low disposable income, lack of health insurance); rural residence; female sex; initial consultation with traditional healers and resource constraints within the health care service [13]. However, to our knowledge, the impact of ethnic minority status upon diagnosis and treatment delays within China’s TB patient population has not been investigated.

China has a unique socio-cultural environment, and understanding this within the context of delays in TB management could be of value in combating the disease [13]. Hunan Province, located in south-central China, is one of the most populous divisions of the country where ethnic minority groups represent 10.1 percent of the population [14]. Despite significant investments in TB control and treatment strategies by the Hunan government [14], which have reduced the burden of disease [15], Hunan remains a high TB burden province. [16, 17].

This study aimed to evaluate the impact of ethnic minority status on the time to diagnosis and the time to treatment among patients registered in Hunan Province between 2013 and 2018.

## Methods

### Study design and data sources

Operating under the provincial health committee, the Hunan Tuberculosis Control Institute is responsible for the province’s TB control and prevention, and research and development [18]. This is a retrospective cohort study conducted on patients diagnosed with pulmonary and extrapulmonary TB in Hunan Province between 2013 and 2018 inclusive. Data were obtained from the internet-based TB management system administered by the TB Control Institute of Hunan Province (TBCIHP).

The date of symptom onset and the date of any previous diagnosis (if any) were recorded in the system on the basis of information provided by the patient. The date of TB diagnosis and date of treatment commencement were recorded by health professionals at the designated TB institutions. Demographic data e.g., ethnic group, sex, age, occupation, year of registration at the designated TB institution and residential address were also available.

### Definitions

**Fig. 1:**
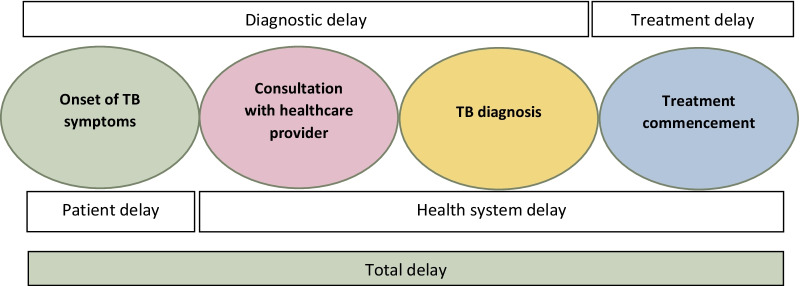
Conceptual framework for TB diagnosis and treatment delay [19]. Total delay = time between the onset of symptoms and the commencement of treatment. Diagnosis delay = time between onset of symptoms and diagnosis. Treatment delay = time between diagnosis and treatment commencement. Patient delay = time between onset of symptoms and consultation with healthcare provider. Health care system delay = time between consultation and treatment commencement

Total delay is defined as the timeframe between the onset of disease and the start of treatment [19]. The total delay can be classified in two ways- as the sum of the diagnosis delay (time between the onset of symptoms and diagnosis) and the treatment delay (time between diagnosis and treatment commencement) or as the patient delay (time between onset of symptoms and consultation with a health care provider) and the health system delay (time between patient consultation and start of treatment, Fig. 1) [19]. This study evaluated diagnostic and treatment delays.

Hunan’s TB institutions follow a TB diagnosis based on WHO recommended methods, e.g., clinical assessment based on symptoms, sputum smear microscopy, chest x-ray, sputum culture and molecular detection [20].

China’s ethnic classification system recognizes 55 minority groups in addition to the Han majority [21]. For this study, associations between diagnosis delay and treatment delay with Tujia, Miao, Dong, Yao, Bai, Mongolian and ‘other’ ethnic minority group status were estimated relative to the Han majority. The ‘other’ ethnic minority group comprised the summation of all other ethnic minority groups, who constitute < 0.1% of the patient population. The ‘other’ group included Buyi, Dai, Gelao, Hani, Hui, Jingpo, Kazakh, Kirgiz, Korean, Lahu, Li, Lisu, Manchu, Salar, She, Tibetan, Tu, Uighur, Wa, Yao, Yi, and Zhuang ethnic minorities.

Definitions pertaining to the other clinical descriptors/variables analyzed are detailed in Table 1.

**Table 1:** Definitions of clinical variables included in the study

Variable/Demographic descriptor	Definition
Residential address	
Local	Patients who reside in local counties
Intra-provincial	Patients who reside in other counties within the province
Inter-provincial	Patients who reside in provinces other than Hunan
Foreign nationality	Patients who reside in other countries
Patient enrolment classification	
Consultation due to symptoms	Patients who consult the TB institution due to symptoms
Referral	Patients who are referred to the TB institution due to symptoms
Contact tracing	TB patients identified by contact tracing
Health check	TB patients who are identified as a result of a health check
TB diagnosis results	
Etiological examine negative	TB cases identified on the basis of symptoms
Smear positive	Positive Acid-Fast Bacillus test
Extrapulmonary TB	TB identified in organs other than the lungs
Culture positive	TB positive sputum culture
Severely ill	Patients with miliary TB, cavities, TB empyema or serious damage to one or more organ caused by TB disease
Drug resistance pattern	
Drug susceptible TB	*M.tuberculosis* that is susceptible to first line antibiotics (isoniazid, rifampin, ethambutol, and pyrazinamide)
MDR-TB	*M.tuberculosis* that is resistant to isoniazid and rifampicin
Mono-resistant TB	*M.tuberculosis* resistant to a single first line antibiotic
Diagnosis institution	
CDC	Centre for disease control and prevention that has a TB clinic
Hospital	General hospital
TB dispensary	Specialized TB hospital (TB patients only)
Other	Other health institution or hospital not covered by above classifications
Registration category	
New patient	TB patients who have never taken anti-TB drugs, or who have been receiving irregular treatment for less than one month
Relapse	TB patients with a history of disease, who complete a full course of chemotherapy and appear cured according to symptoms, but who return a smear positive sputum sample
Return after default	TB patients who receive chemotherapy for ≥ 1 month but discontinue therapy for ≥ 2 months and then return for treatment
Initial treatment failed	New sputum smear positive TB patients with positive sputum smear microscopy results at the end of the 5th month or after completion of therapy; and sputum smear negative TB patients with a positive smear result for any sputum sample
Chronic patient	Positive sputum examination results after several episodes of irregular therapy
Treatment category	
Initial treatment	TB patients who have never taken anti-TB drugs
Retreatment	Patient who has history of TB treatment
TB treatment	
Accept treatment	Patient who accepts the recommended treatment regime
Reject treatment	Patient who rejects the recommended treatment regime

### Statistical analysis

Data were translated from Mandarin to English, checked for completeness, cleaned, and entered into STATA version 16.1 (StataCorp, College Station, TX) for analysis. Frequency and cross-tabulation were used to cross check data completeness.

Descriptive statistics were used to summarize data and define characteristics of the patient population. Treatment and diagnosis delays were calculated in days and summarized, using the median and interquartile range (IQR) because the data showed a non-normal distribution.

To dichotomize data, the median (21 days) was used to define diagnosis delay and the upper quartile (15 days) was used to define treatment delay. Categorical variables were described by counts and percentages, and continuous and normally distributed variables were summarized by means and standard deviations (SD). Univariable logistic regression models were fitted and Crude Odds Ratios (COR) with 95% Confidence Intervals (CI) reported. Multicollinearity between independent variables was assessed using variance inflation factors (VIF) and variables with VIF > 5 were excluded from the final multivariable analysis.

All variables assessed in the univariable models were fitted into multivariable logistic regression models. Adjusted odds ratios and 95% confidence intervals (CI) were computed to measure the association between the dependent (i.e., diagnosis and treatment delays) and independent variables (i.e., ethnic minority status, sex, occupation year of patient registration, residential address, patient enrolment classification, diagnosis institution and whether a patient was severely ill). Variables with a *p*-value < 0.05 in the multivariable analysis were considered as having a statistically significant association with the outcome (diagnosis or treatment delay). Additional models were created for sensitivity analyses to evaluate a 14 day delay used by some studies [13, 22], compared a 21 day diagnosis delay used by others [23–27]. An analysis was also conducted using the median (> 1 day) to define treatment delay.

To evaluate the outcome variables (i.e., diagnosis and treatment delay) in their continuous form sensitivity analyses were undertaken using a negative binomial regression model.

An additional model was constructed to determine treatment delay for patients with two TB diagnosis dates. ‘New patient’ treatment delay was defined as the time period (days) between the date of the second diagnosis and treatment commencement. This analysis was undertaken to mitigate the risk of treatment being administered between a patients first diagnosis and a subsequent diagnosis, a timeframe for which we had no data.

### Ethics statement

Ethical clearance was obtained from Curtin University (HRE2019-0581) and written permission to access the data granted from TBCIHP. Medical records of the patient population were de-identified to preserve privacy. Because this study used secondary, de-identified data, informed patient consent was not required.

## Results

### Socio-demographic and clinical characteristics of the patients

A total of 318,792 TB patients registered in Hunan Province between 2013 and 2018 were included in this study. The sociodemographic characteristics of the patients are presented in Table 2. The majority of patients were male (72.6%) and the study population had a mean age of 51.75 years (SD 17.67). Patients of Han ethnicity formed the majority (90.6%) with the remainder of the patient population represented by 28 ethnic minority groups (ethnicity data were not available for 4 patients). Seventy-eight percent of patients were employed in the agricultural sector. Most patients were new (95.7%), with the majority not severely ill (96.2%) and in receipt of a drug susceptible TB diagnosis (87.2%).

**Table 2:** Sociodemographic and clinical characteristics of TB patients registered in Hunan Province, China, 2013–2018

Variable	Number	Percent
Sex		
Male	231,495	72.62
Female	87,297	27.38
Age (years)		
Mean = 51.75; SD 17.67		
0–10	346	0.11
11–20	15,767	4.95
21–30	37,135	11.65
31–40	30,470	9.56
41–50	56,269	17.65
51–60	62,606	19.64
61–70	69,209	21.71
71–80	38,807	12.17
81–101	8183	2.57
Occupation		
Agriculture^~^	249,093	78.14
Housekeeping^$^	30,802	9.66
Education^∆^	10,679	3.35
Commercial services/civil servant	7818	2.45
Migrant worker	2601	0.82
Healthcare	1009	0.32
Hospitality	612	0.19
Other	16,178	5.07
Ethnicity		
Han	288,802	90.59
Tujia	13,680	4.29
Miao	8460	2.65
Dong	4033	1.27
Yao	2662	0.84
Bai	509	0.16
Mongolian	349	0.11
Other*	293	0.09
Residential address		
Local	310,343	97.35
Intra-provincial	6215	1.95
Inter-provincial	2182	0.68
Foreign nationality	52	0.02
Patient enrolment classification		
Consultation due to symptoms	117,834	36.96
Referral	103,261	32.39
Contact tracing	93,183	29.23
Health check	3179	1.00
Other	1335	0.42
TB diagnosis results		
Etiological examination negative	189,129	59.32
Smear positive	122,006	38.27
Extrapulmonary TB	5609	1.76
Culture positive	1355	0.43
Molecular diagnosis positive	693	0.22
Severely ill		
No	306,534	96.15
Yes	12,258	3.85
Drug resistance pattern		
Drug susceptible TB	15,555	87.23
MDR-TB	1248	7.00
Mono-resistant TB	1030	5.78
Diagnosis institution		
Centre for Disease Control & Prevention (CDC)	278,707	88.15
Hospital	33,104	10.47
TB dispensary	4276	1.35
Other	69	0.02
Registration category		
New patient	305,218	95.74
Relapse	12,179	3.82
Return after default	350	0.11
Initial treatment failed	279	0.09
Chronic patient	122	0.04
Other	644	0.20
Treatment category		
Initial treatment	305,306	95.77
Retreatment	13,486	4.23
TB treatment		
Accept treatment	318,324	99.86
Reject treatment	462	0.17

^~^Agriculture includes famer, herdsman, fisherman

^$^Housekeeping includes housekeeping, childcare, retired and unemployed

^∆^Education includes students and teachers

^*^Other are represented by: Buyi, Dai, Gelao, Hani, Hui, Jingpo, Kazakh, Kirgiz, Korean, Lahu, Li, Lisu, Manchu, Salar, She, Tibetan, Tu, Uighur, Wa, Yao, Yi, and Zhuang ethnic groups

### Median time to diagnosis and treatment by ethnic minority status

Table 3 illustrates median time to diagnosis and treatment by study characteristics. Across all patients, the median time to diagnosis was 21 days (IQR 7–50 days), and the median time to treatment was 1 day (IQR 0–15 days).

**Table 3:** Median time from symptom onset to diagnosis and from diagnosis to treatment commencement for TB patients registered in Hunan Province, China 2013–2018, by demographic and clinical characteristics

	Number of patients (%)	Median time to diagnosis (days)	Median time to treatment (days)
All patients	318,792	21 (IQR 7–50)	1 (IQR 0–15)
Ethnicity			
Han	288,802 (90.59)	20 (IQR 6–49)	1 (IQR 0–16)
Tujia	13,680 (4.29)	30 (IQR 7– 65)	1 (IQR 0–9)
Miao	8460 (2.65)	27 (IQR 10–61)	1 (IQR 0–9)
Dong	4033 (1.27)	35 (IQR 10–75)	1 (IQR 0–9)
Yao	2662 (0.84)	24 (IQR 7–58)	0 (IQR 0–2)
Bai	509 (0.16)	28 (IQR 7–51)	2 (IQR 0–9)
Mongolian	349 (0.11)	23 (IQR 7–52)	1 (IQR 0–12)
Other*	293 (0.09)	16 (IQR 3–46)	1 (IQR 0–9)
Sex			
Male	231,495(72.62)	21 (IQR 7–50)	1 (IQR 0–14)
Female	87,297 (27.38)	21 (IQR 7–51)	1 (IQR 0–17)
Age			
< 18 years	7155 (2.24)	14 (IQR 3–36)	2 (IQR 0–17)
> 18 years	311,637 (97.76)	21 (IQR 7–51)	1 (IQR 0–15)
Occupation			
Agriculture^~^	249,093 (78.14)	22 (IQR 7–54)	1 (IQR 0–12)
Housekeeping^$^	30,802 (9.66)	19 (IQR 5–46)	4 (IQR 0–27)
Education^∆^	10,679 (3.35)	13 (IQR 3–33)	2 (IQR 0–20)
Commercial services/civil servant	7818 (2.45)	16 (IQR 4–39)	7 (IQR 0–30)
Migrant worker	2601 (0.82)	19 (IQR 7–46)	1 (IQR 0–10)
Healthcare	1009 (0.32)	15 (IQR 4–36)	4 (IQR 0–25)
Hospitality	612 (0.19)	14 (IQR 3–32)	6 (IRQ 0–27)
Other	16,178 (5.07)	16 (IQR 4–40)	2 (IQR 0–23)
Year			
2013	56,198 (17.63)	21 (IQR 6–55)	1 (IRQ 0–14)
2014	55,815 (17.51)	21 (IQR 7–51)	1 (IQR 0–14)
2015	55,196 (17.31)	21 (IQR 7–50)	1 (IQR 0–14)
2016	49,996 (15.68)	22 (IQR 7–52)	1 (IQR 0–13)
2017	49,843 (15.63)	21 (IQR 6–48)	1 (IQR 0–16)
2018	51,744 (16.23)	19 (IQR 6–48)	1 (IQR 0–18)
Residential address			
Local	310,343 (97.35)	21 (IQR 7–50)	1 (IQR 0–14)
Intra-provincial (within province)	6215 (1.95)	27 (IQR 6–59)	2 (IQR 0–30)
Inter-provincial (between provinces)	2182 (0.68)	18 (IQR 4–44)	5 (IQR 0–28)
Foreign nationality	52 (0.02)	29.5 (IQR 7.5–65)	0 (IQR 0–4)
Patient enrolment classification			
Consultation due to symptoms	117,834 (36.96)	26 (IQR 11–60)	0 (IQR 0–1)
Referral	103,261 (32.39)	17 (IQR 5–45)	1 (IQR 0–12)
Contact tracing	93,183 (29.23)	19 (IQR 4–48)	17 (IQR 0–38)
Health check	3179 (1.00)	3 (IQR 0–14)	0 (IQR 0–3)
Other	1335 (0.42)	15 (IQR 4–34)	8 (IQR 1–28)
Diagnosis institution			
CDC	278,707 (88.15)	21 (IQR 7–51)	1 (IQR 0–15)
Hospital	33,104 (10.47)	20 (IQR 6–45)	0 (IQR 0–9)
TB dispensary	4276 (1.35)	19 (IQR 10–35)	11 (IQR 2–18)
Other	69 (0.02)	16 (IQR 5–38)	1 (IQR 0–7)
Severely ill			
No	306,534 (96.15)	21 (IQR 7–50)	1 (IQR 0–15)
Yes	12,258 (3.85)	28 (IQR 9–62)	1 (IQR 0–13)

^~^Agriculture includes famer, herdsman, fisherman

^$^Housekeeping includes housekeeping, childcare, retired and unemployed

^∆^Education includes students and teachers

^*^Other are represented by: Buyi, Dai, Gelao, Hani, Hui, Jingpo, Kazakh, Kirgiz, Korean, Lahu, Li, Lisu, Manchu, Salar, She, Tibetan, Tu, Uighur, Wa, Yao, Yi, and Zhuang ethnic groups

Results show differences in median time to diagnosis and treatment across different ethnic groups. The median time to diagnosis for the Han majority population was 20 days (IQR 6–49 days); 30 days (IQR 7–65 days) for Tujia; 27 days (IQR 10–61 days) for Miao; 35 days (IQR 10–75 days) for Dong; 24 days (IQR 7–58 days) for Yao; 28 days (IQR 7–51 days) for Bai; 23 days (IQR 7–52 days) for Mongolian and 16 days (IQR 3–46 days) for ‘other’ ethnic minority groups. For each of the ethnic groups, the median time to diagnosis by year of patient registration is represented graphically in Fig. 1 of the Additional file 1. The median time to treatment was 1 day (IQR 0–16 days) for Han; 1 day (IQR 0–9 days) for Tujia; 1 day (IQR 0–9 days) for Miao; 1 day (IQR 0–9 days) for Dong; 0 days (IQR 0–2 days) for Yao; 2 days (IQR 0–9 days) for Bai; 1 day (IQR 0–12 days) for Mongolian and 1 day (IQR 0–9 days) for ‘other’ ethnic minority groups.

The median time to diagnosis (21 days) was used to define delay in subsequent analyses. For clinical relevance, the upper quartile (15 days) was used to define treatment delay, with a sensitivity analysis conducted at the median (1 day).

### Factors associated with tuberculosis diagnosis delays

Results of univariable and multivariable logistic regression models to identify factors associated with diagnosis delay are detailed in Table 4. Univariable analysis shows five of the seven ethnic minority groups (i.e., Tujia, Miao, Dong, Yao, and Bai) to have significantly longer diagnosis delays than the reference Han majority. The same five ethnic minority groups had significant greater odds of experiencing diagnosis delays in the multivariable models. The odds of experiencing diagnosis delays relative to the Han majority were significantly higher for Tujia (adjusted odds ratio (AOR): 1.46, 95% CI: 1.41, 1.51), Miao (AOR: 1.31, 95% CI: 1.26, 1.37), Dong (AOR: 1.97, 95% CI: 1.85, 2.11), Yao (AOR: 1.27, 95% CI: 1.17, 1.37), and Bai (AOR: 1.45, 95% CI: 1.22, 1.74) ethnic minorities. Differences in diagnosis delay for the Mongolian ethnic group (AOR 1.20, 95% CI 0.97, 1.48) and the ‘other’ ethnic minorities (AOR 0.92 95% CI 0.73, 1.17) relative to the Han majority were not significant.

**Table 4:** Univariable and multivariable regression assessment of factors associated with 21 day diagnosis delay in TB patients registered in Hunan Province, 2013–2018

	Number of patients (%)	Univariable odds ratio (95% CI)	Univariable *p* value	Multivariable odds ratio (95% CI)	Multivariable *p* value
Ethnicity					
Han	288,802 (90.59)	1.00		1.00	
Tujia	13,680 (4.29)	1.38 (1.33, 1.43)	0.000	1.46 (1.41, 1.51)	0.000
Miao	8460 (2.65)	1.29 (1.77, 2.02)	0.000	1.31 (1.26, 1.37)	0.000
Dong	4033 (1.27)	1.89 (1.77, 2.02)	0.000	1.97 (1.85, 2.11)	0.000
Yao	2662 (0.84)	1.18 (1.10, 1.28)	0.000	1.27 (1.17, 1.37)	0.000
Bai	509 (0.16)	1.30 (1.09, 1.55)	0.004	1.45 (1.22, 1.74)	0.000
Mongolian	349 (0.11)	1.21 (0.98, 1.49)	0.078	1.20 (0.97, 1.48)	0.099
Other*	293 (0.09)	0.81 (0.64, 1.02)	0.067	0.92 (0.73, 1.17)	0.494
Sex					
Male	231,495 (72.62)	1.00		1.00	
Female	87,297 (27.38)	1.02 (1.00, 1.03)	0.021	1.04 (1.03, 1.06)	0.000
Age	318,792 (100)	1.01 (1.01, 1.01)	0.000	1.004 (1.003, 1.004)	0.000
Occupation					
Commercial services/civil servant	7818 (2.45)	1.00		1.00	
Agriculture ~	249,093 (78.14)	1.42 (1.36, 1.49)	0.000	1.25 (1.19, 1.31)	0.000
Housekeeping^$^	30,802 (9.66)	1.23 (1.17, 1.30)	0.000	1.17 (1.11, 1.23)	0.000
Education^∆^	10,679 (3.35)	0.80 (0.75, 0.85)	0.000	0.84 (0.79, 0.90)	0.000
Migrant worker	2601 (0.82)	1.17 (1.07, 1.28)	0.000	1.06 (0.97, 1.16)	0.196
Healthcare	1009 (0.32)	0.96 (0.84, 1.10)	0.556	0.94 (0.82, 1.07)	0.332
Hospitality	612 (0.19)	0.80 (0.67, 0.94)	0.009	0.82 (0.69, 0.97)	0.022
Other	16,178 (5.07)	1.06 (1.01, 1.12)	0.027	0.98 (0.93, 1.04)	0.508
Year					
2013	56,198 (17.63)	1.00		1.00	
2014	55,815 (17.51)	1.01 (0.99, 1.04)	0.278	0.99 (0.97, 1.02)	0.648
2015	55,196 (17.31)	1.01 (0.99, 1.03)	0.428	0.99 (0.97, 1.02)	0.603
2016	49,996 (15.68)	1.07 (1.04, 1.10)	0.000	1.06 (1.03, 1.09)	0.000
2017	49,843 (15.63)	1.00 (0.97, 1.02)	0.798	1.01 (0.98, 1.03)	0.550
2018	51,744 (16.23)	0.93 (0.91, 0.96)	0.000	1.01 (0.98, 1.03)	0.656
Residential address					
Local	310,343 (97.35)	1.00		1.00	
Intra-provincial (within province)	6215 (1.95)	1.23 (1.17, 1.30)	0.000	1.48 (1.41, 1.56)	0.000
Inter-provincial (between provinces)	2182 (0.68)	0.86 (0.79, 0.94)	0.001	1.06 (0.98, 1.16)	0.153
Foreign nationality	52 (0.02)	1.45 (0.83, 2.53)	0.186	1.58 (0.90, 2.79)	0.111
Patient enrolment classification					
Consultation due to symptoms	117,834 (36.96)	1.00		1.00	
Referral	103,261 (32.39)	0.66 (0.65, 0.67)	0.000	0.65 (0.63, 0.66)	0.000
Contact tracing	93,183 (29.23)	0.73 (0.72, 0.74)	0.000	0.74 (0.72, 0.75)	0.000
Health check	3179 (1.00)	0.18 (0.17, 0.20)	0.000	0.20 (0.18, 0.22)	0.000
Other	1335 (0.42)	0.53 (0.47, 0.59)	0.000	0.55 (0.49, 0.61)	0.000
Diagnosis Institution					
CDC	278,707 (88.15)	1.00		1.00	
Hospital	33,104 (10.47)	0.92 (0.90, 0.95)	0.000	0.93 (0.91, 0.96)	0.000
TB dispensary	4276 (1.35)	0.74 (0.69, 0.78)	0.000	0.72 (0.67, 0.76)	0.000
Other	69 (0.02)	0.75 (0.46, 1.20)	0.226	0.85 (0.52, 1.39)	0.521
Severely Ill					
No	306,534 (96.15)	1.00		1.00	
Yes	12,258 (3.85)	1.31 (1.00, 1.02)	0.000	1.35 (1.31, 1.41)	0.000

^~^Agriculture includes famer, herdsman, fisherman

^$^Housekeeping includes housekeeping, childcare, retired and unemployed

^∆^Education includes students and teachers

^*^Other are represented by: Buyi, Dai, Gelao, Hani, Hui, Jingpo, Kazakh, Kirgiz, Korean, Lahu, Li, Lisu, Manchu, Salar, She, Tibetan, Tu, Uighur, Wa, Yao, Yi, and Zhuang ethnic groups

The results of the sensitivity analysis using > 14 days to define a diagnosis delay are presented in the Additional file (Additional file 1: Table S1). The analysis shows there to be no difference (14 day vs. 21 day) in the ethnic minority groups that are associated with a significant diagnosis delay relative to the Han majority.

Other variables found to be associated with a > 21 day diagnosis delay in the multivariable analysis include female sex (AOR: 1.04; 95% CI 1.03,1.06); increasing age (AOR 1.004 per one year increase; 95% CI 1.003, 1.004); agriculture (AOR 1.25; 95% CI 1.19, 1.31) and housekeeping (AOR 1.17; 95% CI 1.11, 1.23) occupations relative to the commercial services/civil servants; patient registrations in 2016 (AOR 1.06, 95% CI 1.03, 1.09) relative to 2013; residing within the province (AOR 1.48; 95% CI 1.41, 1.56) relative to being local; and being severely ill (AOR 1.35; 95% CI 1.31, 1.41).

The negative binomial regression assessment of factors associated with time to diagnosis is detailed in the Additional file (Additional file 1: Table S2).

### Factors associated with tuberculosis treatment delays

Results of univariable and multivariable regression models to identify factors associated with treatment delay > 15 days are detailed in Table 5. The multivariable analysis shows that five of the seven ethnic minority groups have significantly lower odds of treatment delay than the Han majority: Tujia (AOR 0.92, 95% CI 0.88, 0.96), Miao (AOR 0.74, 95% CI 0.70, 0.79), Dong (AOR 0.87, 95% CI 0.81, 0.95), Yao (AOR 0.20, 95% CI 0.17, 0.24) and ‘other’ (AOR 0.70, 95% CI 0.51, 0.97).

**Table 5:** Univariable and multivariable regression of factors associated with 15 day treatment delay in TB patients registered in Hunan Province, 2013–2018

	Univariable odds ratio (95% CI)	Univariable *p* value	Multivariable odds ratio (95% CI)	Multivariable *p* value
Ethnicity				
Han	1.00		1.00	
Tujia	0.75 (0.71, 0.78)	0.000	0.92 (0.88, 0.96)	0.000
Miao	0.61 (0.58, 0.65)	0.000	0.74 (0.70, 0.79)	0.000
Dong	0.77 (0.72, 0.84)	0.000	0.87 (0.81, 0.95)	0.001
Yao	0.20 (0.17, 0.24)	0.000	0.20 (0.17, 0.24)	0.000
Bai	0.67 (0.54, 0.84)	0.000	0.83 (0.65, 1.05)	0.126
Mongolian	0.85 (0.66, 1.09)	0.205	0.78 (0.59, 1.03)	0.082
Other*	0.69 (0.52, 0.93)	0.014	0.70 (0.51, 0.97)	0.030
Sex				
Male	1.00		1.00	
Female	1.11 (1.09, 1.13)	0.000	1.07 (1.05, 1.09)	0.000
Age	0.998 (0.998, 0.999)	0.000	1.001 (1.0004, 1.002)	0.000
Occupation				
Commercial services/civil servant	1.00		1.00	
Agriculture ~	0.45 (0.43, 0.47)	0.000	0.58 (0.55, 0.61)	0.000
Housekeeping^$^	0.85 (0.81, 0.90)	0.000	0.81 (0.76, 0.86)	0.000
Education^∆^	0.64 (0.60, 0.68)	0.000	0.67 (0.63, 0.72)	0.000
Migrant worker	0.41 (0.37, 0.46)	0.000	0.64 (0.57, 0.72)	0.000
Healthcare	0.80 (0.70, 0.92)	0.002	0.81 (0.70, 0.95)	0.009
Hospitality	0.87 (0.73, 1.03)	0.115	0.91 (0.75, 1.10)	0.320
Other	0.68 (0.64, 0.72)	0.000	0.82 (0.77, 0.87)	0.000
Year				
2013	1.00		1.00	
2014	0.98 (0.96, 1.01)	0.266	0.94 (0.91, 0.97)	0.000
2015	0.97 (0.94, 0.99)	0.026	0.93 (0.90, 0.96)	0.000
2016	0.94 (0.91, 0.96)	0.000	0.89 (0.87, 0.93)	0.000
2017	1.06 (1.03, 1.09)	0.000	0.98 (0.95, 1.02)	0.309
2018	1.17 (1.14, 1.20)	0.000	1.08 (1.05, 1.12)	0.000
Residential address				
Local	1.00		1.00	
Intra-provincial	1.74 (1.65, 1.84)	0.000	0.97 (0.91, 1.02)	0.233
Inter-provincial	1.74 (1.60, 1.90)	0.000	1.14 (1.04, 1.26)	0.008
Foreign nationality	0.75 (0.38, 1.50)	0.418	1.18 (0.56, 2.51)	0.660
Patient enrolment				
Consult-symptoms	1.00		1.00	
Referral	3.62 (3.53, 3.72)	0.000	3.62 (3.52, 3.72)	0.000
Contact tracing	14.44 (14.07, 14.83)	0.000	14.45 (14.06, 14.84)	0.000
Health check	1.15 (1.00, 1.31)	0.042	1.10 (0.96, 1.25)	0.181
Other	8.38 (7.49, 9.39)	0.000	7.78 (6.94, 8.72)	0.000
Diagnosis institution				
CDC	1.00		1.00	
Hospital	0.74 (0.72, 0.76)	0.000	0.72 (0.69, 0.74)	0.000
TB dispensary	1.40 (1.31, 1.49)	0.000	3.32 (3.09, 3.56)	0.000
Other	0.64 (0.34, 1.19)	0.156	0.78 (0.39, 1.55)	0.477
Severely Ill				
No	1.00		1.00	
Yes	0.86 (0.83, 0.90)	0.000	0.73 (0.69, 0.76)	0.000

^~^Agriculture includes famer, herdsman, fisherman

^$^Housekeeping includes housekeeping, childcare, retired and unemployed

^∆^Education includes students and teachers

^*^Other are represented by: Buyi, Dai, Gelao, Hani, Hui, Jingpo, Kazakh, Kirgiz, Korean, Lahu, Li, Lisu, Manchu, Salar, She, Tibetan, Tu, Uighur, Wa, Yao, Yi, and Zhuang ethnic groups

A sensitivity analysis using the median (> 1 day) to define treatment delay is presented in the Additional file (Additional file 1: Table S3). This analysis shows a variety of treatment delays across the different ethnic groups, with no clear trend detectable.

The other variables associated with > 15 day treatment delay in the multivariable model include female sex (AOR 1.07; 95% CI 1.05, 1.09); increasing age (AOR 1.001 per one year increase; 95% CI 1.0004, 1.002); 2018 as the year of registration relative to 2013 (AOR 1.08; 95% CI 1.05, 1.12); residing inter-provincially relative to being local (AOR 1.14; 95% CI 1.04, 1.26); being enrolled due to referral (AOR 3.62, 95% CI 3.52, 3.72), contact tracing (AOR 14.45, 95% CI 14.06, 14.84) and for other reasons (AOR 7.78, 95% CI 6.94, 8.72) relative to consultation due to symptoms and being diagnosed at a TB dispensary (AOR 3.32; 95% CI 3.09, 3.56) relative to a CDC.

The negative binomial regression assessment of factors associated time from diagnosis to treatment commencement is detailed in the Additional file (Additional file 1: Table S4).

New patients represented 95.74% of the total study population and the results of the sensitivity analysis showed that there were no differences in treatment delays across the study variables between the two population groups (i.e., all TB patients vs. new patients only) (S1 Tables 5 and 6).

## Discussion

Within Hunan Province, this study shows consistent and significant diagnosis delays for ethnic minority TB patients compared to the Han majority. However ethnic minority groups have lower odds of treatment delay relative to the Han majority.

Variables associated with TB diagnosis and treatment delay in previous studies include poverty, socio-economic disadvantage, knowledge, cultural beliefs, literacy, language, and distance and cultural barriers to health care provision [28–31]. The significant difference in the odds of TB diagnosis delay observed between Han majority and ethnic minority patients, and the differences observed between ethnic minority groups, may in part reflect socio-economic and cultural differences that have been reported to be associated with delay by previous studies. [28–31].

The ethnic minorities that inhabit Hunan occupy 28% of the province’s land area [14], with approximately 96% occupying six cities and prefectures located within the ‘Great Western Hunan’ region [32], a region that is rural and less developed. In many rural and remote areas of China there is a lack of infrastructure and resources and a disparity in accessibility to services and facilities. [33, 34].

Although the disparity between urban and rural incomes in China is reducing, in 2019 the respective ratio was 2.59:1 in Hunan Province [35]. Disposable income is an important metric as TB patients face a myriad of direct (e.g., out of pocket medical expenses and health insurance exclusions/co-payments) and indirect costs (e.g., loss of income, cost of transport, food and accommodation) [7, 36–38]. Despite China successfully progressing its goal of universal public health insurance, catastrophic health expenditure (CHE) continues to be a significant confounder in effective TB diagnosis and treatment outcomes [9, 36, 39, 40]. 2016 figures estimate 15.11% of Chinese households experience CHE, with the rate 1.36 times higher in rural compared to urban households [41].

One of the most important confounders in the Chinese urban–rural income gap is education [42]. Improving ethnic minority educational attainment has been a high priority for China since 1949, prior to which it is estimated that up to 80% of its minority population were illiterate [43]. Despite the implementation of preferential policies however, lagging educational attainment continues to contribute to the Han-minority opportunity gap [44]. In addition to the differential between minority and majority populations, there is significant variation in educational attainment between minority groups [45].

The findings of this study show there are opportunities to reduce diagnosis delay within ethnic minority populations. The data supports integration of TB screening within routine health checks, a process that has been shown to be cost effective at improving case detection [46]. As evidenced by other studies, opportunities to improve patient seeking behaviour may relate to the socio-economic and cultural disadvantage experienced by ethnic minorities [13]. Health literacy is a key component of health seeking behaviour thereby reducing diagnosis delay. Population surveys in China show rural location and illiteracy to be significant risk factors in understanding TB and its symptoms [47]. Health seeking behaviour is also impacted by awareness of the NTP and distance to the nearest hospital [48]. Due to structural and economic constraints, patients in rural locations usually seek initial care within their own communities which often adds to the time delay in receiving a correct diagnosis [49].TB health seeking behaviour is also impacted by stigma of the disease, which in itself it impacted by social and cultural context [49, 50]. Due to the significant diversity between and within different Chinese ethnic groups [44], detailed socioeconomic and cultural information is required to inform appropriate interventions.

When evaluating treatment delay, this study found all ethnic minority groups had lower odds of delay than the Han majority, with the finding significant in five of the seven ethnic minority groups. Further research is required to elucidate why the majority population is at greater risk of a treatment delay, and whether these findings are attributable to success of the NTP which prioritizes vulnerable population groups [9, 10]. Another possible explanation is that due to diagnosis delay in ethnic minorities, an increase in disease progression may lead to prioritization of treatment, however further research is required to test this hypothesis.

A significant strength of this study is the large, detailed data set on a well described cohort of patients. However, a lack of information on variables that may relate to the underlying causes of delay e.g., income and level of education, is a limitation of this study. The study is reliant upon the patient for the date of symptom onset from which diagnosis and treatment delay are calculated. Recall bias on the date of symptom onset therefore has the potential to impact subsequent findings. Only patients presenting for treatment at designated institutions are included in the analysis and so the data may not be representative of the variables across all TB patients within the province.

## Conclusions

Reducing the time between TB onset and treatment is important in reducing morbidity and mortality and preventing further disease transmission This study shows ethnic minority groups experience significant TB diagnosis delay compared to the Han majority. Ethnicity is a complex variable that is often associated with a multitude of socio-economic disparities. These disparities are likely to be the underlying root cause of TB delay differentials observed between and within different population groups, which highlights the need for further research. It is also recommended that further studies evaluate the impact of ethnicity on TB treatment outcomes, as treatment outcomes are also key to effective TB control.

## Supplementary Information

Additional file 1: **Fig S1.** Median time to diagnosis by ethnicity for TB patients registered in Hunan Province 2013-2018. **Table S1**. Sensitivity Analysis: Univariable and multivariable regression of factors associated with 14 day diagnosis delay in TB patients registered in Hunan Province, 2013-2018. **Table S2.** Univariable and multivariable negative binomial regression assessment of factors associated with time to diagnosis in TB patients registered in Hunan Province, 2013-2018. **Table S3**. Sensitivity Analysis: Univariable and multivariable regression assessment of factors associated with 1 day treatment delay in TB patients registered in Hunan Province, 2013-2018. **Table S4.** Univariable and multivariable negative binomial regression assessment of factors associated with time from diagnosis to treatment commencement in TB patients registered in Hunan Province, 2013-2018. **Table** **S5**. Median time from diagnosis to treatment commencement for new TB patients registered in Hunan Province, 2013-2018, by demographic characteristics. **Table S6.** Univariable and multivariable regression of factors associated with >15 day treatment delay in new TB patients registered in Hunan Province, 2013-2018.

## Data Availability

The datasets analysed during the current study are available from the corresponding author on reasonable request.

## Abbreviations

AOR: Adjusted odds ratio
CDC: Centre for disease control and prevention
CHE: Catastrophic health expenditure
CI: Confidence interval
COVID-19: Coronavirus disease 2019
DOTS: Directly observed treatment short-course
HIV: Human immunodeficiency virus
IQR: Interquartile range
IQR: Multi-drug resistant
NTP: National Tuberculosis Control Programme
p-value: Probability value
SD: Standard deviation
TB: Tuberculosis
TBCIHP: TB Control Institute of Hunan Province
VIF: Variance inflation factor
WHO: World Health Organization
