# Supplementary material for: The impact of ethnic minority status on tuberculosis diagnosis and treatment delays in Hunan Province, China
Source: BMC Infect Dis. 2022 Jan 26;22:90. doi: 10.1186/s12879-022-07072-4 (PMC8790941; doi:10.1186/s12879-022-07072-4)
Supplement: Supplementary file 1 — Additional file 1: Fig S1. Median time to diagnosis by ethnicity for TB patients registered in Hunan Province 2013-2018. Table S1. Sensitivity Analysis: Univariable and multivariable regression of factors associated with 14 day diagnosis delay in TB patients registered in Hunan Province, 2013-2018. Table S2. Univariable and multivariable negative binomial regression assessment of factors associated with time to diagnosis in TB patients registered in Hunan Province, 2013-2018. Table S3. Sensitivity Analysis: Univariable and multivariable regression assessment of factors associated with 1 day treatment delay in TB patients registered in Hunan Province, 2013-2018. Table S4. Univariable and multivariable negative binomial regression assessment of factors associated with time from diagnosis to treatment commencement in TB patients registered in Hunan Province, 2013-2018. Table S5. Median time from diagnosis to treatment commencement for new TB patients registered in Hunan Province, 2013-2018, by demographic characteristics. Table S6. Univariable and multivariable regression of factors associated with >15 day treatment delay in new TB patients registered in Hunan Province, 2013-2018. [file 12879_2022_7072_MOESM1_ESM.docx]

**Additional file 1**

**Fig S1:** Median time to diagnosis by ethnicity for TB patients registered in Hunan Province 2013-2018

**Table S1**: Sensitivity Analysis: Univariable and multivariable regression of factors associated with 14-day diagnosis delay in TB patients registered in Hunan Province, 2013-2018

|  | Univariable odds ratio (95% CI) | Univariable *p* value | Multivariable odds ratio (95% CI) | Multivariable *p* value |
| --- | --- | --- | --- | --- |
| **Ethnicity**  Han  Tujia  Miao  Dong  Yao  Bai  Mongolian  Other* | 1.00  1.26 (1.22, 1.31)  1.44 (1.37, 1.50)  1.58 (1.48, 1.69)  1.15 (1.06, 1.25)  1.18 (0.99, 1.42)  1.14 (0.92, 1.42)  0.77 (0.61, 0.96) | 0.001  0.000  0.000  0.001  0.068  0.235  0.024 | 1.00  1.34 (1.29, 1.39)  1.48 (1.42, 1.56)  1.68 (1.57, 1.80)  1.24 (1.14, 1.34)  1.33 (1.10, 1.60)  1.13 (0.90, 1.41)  0.88 (0.69, 1.11) | 0.000  0.000  0.000  0.000  0.003  0.296  0.283 |
| **Sex**  Male  Female | 1.00  1.01 (0.997, 1.03) | 0.110 | 1.04 (1.02, 1.06) | 0.000 |
| **Age** | 1.01 (1.01, 1.01) | 0.000 | 1.00 (1.003, 1.004) | 0.000 |
| **Occupation**  Commercial services/civil servant  Agriculture~  Housekeeping^$^  Education^∆^  Migrant worker  Healthcare  Hospitality  Other | 1.00  1.47 (1.40, 1.53)  1.25 (1.19, 1.32)  0.81 (0.76, 0.86)  1.40 (1.27, 1.53)  1.00 (0.88, 1.14)  0.87 (0.74, 1.03)  1.07 (1.01, 1.13) | 0.000  0.000  0.000  0.000  0.971  0.098  0.017 | 1.00  1.23 (1.17, 1.29)  1.18 (1.12, 1.24)  0.85 (0.80, 0.90)  1.19 (1.09, 1.31)  0.96 (0.84, 1.10)  0.89 (0.75, 1.05)  0.96 (0.90, 1.01) | 0.000  0.000  0.000  0.000  0.554  0.170  0.112 |
| **Year**  2013  2014  2015  2016  2017  2018 | 1.00  1.04 (1.01, 1.06)  1.06 (1.04, 1.09)  1.13 (1.10, 1.16)  1.00 (0.98, 1.03)  0.92 (0.90, 0.95) | 0.005  0.000  0.000  0.777  0.000 | 1.00  1.02 (0.99, 1.04)  1.05 (1.02, 1.07)  1.11 (1.09, 1.14)  1.01 (0.99, 1.04)  0.99 (0.96, 1.01) | 0.144  0.000  0.000  0.340  0.311 |
| **Residential Address**  Local  Intra-provincial  Inter-provincial  Foreign nationality | 1.00  1.12 (1.07, 1.19)  0.80 (0.74, 0.87)  1.17 (0.66, 2.08) | 0.000  0.000  0.582 | 1.00  1.43 (1.36, 1.51)  1.02 (0.94, 1.12)  1.25 (0.70, 2.25) | 0.000  0.608  0.454 |
| **Patient Classification**  Consultation symptoms  Referral  Contact tracing  Health check  Other | 1.00  0.57 (0.56, 0.58)  0.59 (0.58, 0.60)  0.15 (0.14, 0.16)  0.48 (0.43, 0.54) | 0.000  0.000  0.000  0.000 | 1.00  0.56 (0.55, 0.57)  0.60 (0.59, 0.61)  0.16 (0.15, 0.17)  0.51 (0.45, 0.57) | 0.000  0.000  0.000  0.000 |
| **Diagnosis Institution**  CDC  Hospital  TB dispensary  Other | 1.00  0.96 (0.94, 0.98)  1.31 (1.23, 1.40)  0.91 (0.56, 1.47) | 0.001  0.000  0.700 | 1.00  1.00 (0.97, 1.03)  1.24 (1.16, 1.32)  1.08 (0.66, 1.77) | 0.897  0.000  0.769 |
| **Severely Ill**  No  Yes | 1.00  1.27 (1.22, 1.32) | 0.000 | 1.00  1.33 (1.28, 1.39) | 0.000 |

^~^ Agriculture includes famer, herdsman, fisherman.

^$^ Housekeeping includes housekeeping, childcare, retired and unemployed.

^∆^ Education includes students and teachers.

*Other are represented by: Buyi, Dai, Gelao, Hani, Hui, Jingpo, Kazakh, Kirgiz, Korean, Lahu, Li, Lisu, Manchu, Salar, She, Tibetan, Tu, Uighur, Wa, Yao, Yi, and Zhuang ethnic groups.

**Table S2:** Univariable and multivariable negative binomial regression assessment of factors associated with time to diagnosis in TB patients registered in Hunan Province, 2013-2018

|  | Univariable coefficient  (95% CI) | Univariable *p* value | Multivariable coefficient  (95% CI) | Multivariable *p* value |
| --- | --- | --- | --- | --- |
| **Ethnicity**  Han  Tujia  Miao  Dong  Yao  Bai  Mongolian  Other* | 0.00  0.17 (0.15, 0.20)  0.06 (0.02, 0.09)  0.51 (0.47, 0.56)  -0.06 (-0.12, -0.001)  0.69 (0.56, 0.82)  -0.05 (-0.21, 0.11)  -0.24 (-0.42, -0.07) | 0.000  0.001  0.000  0.046  0.000  0.560  0.007 | 0.00  0.23 (0.20, 0.25)  0.13 (0.09, 0.16)  0.52 (0.47, 0.57)  0.05 (-0.008, 0.11)  0.60 (0.47, 0.74)  -0.07 (-0.24, 0.09)  -0.10 (-0.27, 0.08) | 0.000  0.000  0.000  0.089  0.000  0.367  0.287 |
| **Sex**  Male  Female | 0.00  -0.01 (-0.02, -0.002) | 0.017 | 0.00  0.02 (0.005, 0.03) | 0.005 |
| **Age** | 0.01 (0.01, 0.01) | 0.000 | 0.01 (0.01, 0.01) | 0.000 |
| **Occupation**  Commercial services/civil servant  Agriculture~  Housekeeping^$^  Education^∆^  Migrant worker  Healthcare  Hospitality  Other | 0.00  0.29 (0.26, 0.32)  0.26 (0.22, 0.30)  -0.42 (-0.46, -.037)  0.16 (0.09, 0.22)  -0.41 (-0.51, -0.31)  -0.45 (-0.58, -0.32)  0.003 (-0.04, 0.04) | 0.000  0.000  0.000  0.000  0.000  0.000  0.883 | 0.00  0.13 (0.10, 0.17)  0.10 (0.06, 0.14)  -0.18 (-0.22, -0.13)  0.14 (0.07, 0.21)  -0.34 (-0.44, -0.24)  -0.29 (-0.42, -0.17)  -0.03 (-0.07, 0.01) | 0.000  0.000  0.000  0.000  0.000  0.000  0.142 |
| **Year**  2013  2014  2015  2016  2017  2018 | zero  0.04 (0.02, 0.06)  -0.004 (-0.02, 0.01)  -0.009 (-0.03, 0.009)  -0.06 (-0.07, -0.04)  -0.08 (-0.10, -0.06) | 0.000  0.698  0.337  0.000  0.000 | zero  -0.02 (0.004, 0.04)  -0.02 (-0.04, -0.002)  -0.0009 (-0.02, 0.02)  -0.03 (-0.05, -0.02)  -0.003 (-0.02, 0.02) | 0.016  0.026  0.924  0.000  0.796 |
| **Residential Address**  Local  Intra-provincial (within province)  Inter-provincial (between provinces)  Foreign nationality | 0.00  0.002 (-0.04, 0.04)  -0.31 (-0.38, -0.25)  0.06 (-0.36, 0.47) | .923  0.000  0.794 | 0.00  0.14 (0.10, 0.18)  -0.09 (-0.16, -0.03)  0.29 (-0.13, 0.70) | 0.000  0.006  0.174 |
| **Patient Enrolment Classification**  Consultation due to symptoms  Referral  Contact tracing  Health check  Other | 0.00  -0.09 (-0.10, -0.07)  0.04 (0.03, 0.05)  -1.30 (-1.36, -1.25)  -0.28 (-0.36, -0.19) | 0.000  0.000  0.000  0.000 | 0.00  -0.08 (-0.09, -0.07)  0.05 (0.04, 0.06)  -1.07 (-1.13, -1.02)  -0.28 (-0.36, -0.19) | 0.000  0.000  0.000  0.000 |
| **Diagnosis Institution**  CDC  Hospital  TB dispensary  Other | 0.00  -0.21 (-0.22, -0.19)  -0.23 (-0.28, -0.19)  -0.58 (-0.94, -0.21) | 0.000  0.000  0.002 | 0.00  -0.19 (-0.21, -0.17)  -0.22 (-0.26, -0.17)  -0.31 (-0.67, 0.05) | 0.000  0.000  0.091 |
| **Severely Ill**  No  Yes | 0.00  0.05 (0.02, 0.07) | 0.001 | 0.00  0.05 (0.02, 0.08) | 0.001 |

**Table S3**: Sensitivity Analysis: Univariable and multivariable regression assessment of factors associated with 1-day treatment delay in TB patients registered in Hunan Province, 2013-2018

|  | Number of patients (%) | Univariable odds ratio (95% CI) | Univariable *p* value | Multivariable odds ratio  (95% CI) | Multivariable *p* value |
| --- | --- | --- | --- | --- | --- |
| **Ethnicity**  Han  Tujia  Miao  Dong  Yao  Bai  Mongolian  Other* | 288,802 (90.59)  13,680 (4.29)  8,460 (2.65)  4,033 (1.27)  2,662 (0.84)  509 (0.16)  349 (0.11)  293 (0.09) | 1.00  0.99 (0.95, 1.02)  1.05 (1.00, 1.09)  0.83 (0.78, 0.89)  0.55 (0.51, 0.60)  1.24 (1.05, 1.48)  0.93 (0.75, 1.15)  0.96 (0.76, 1.21) | 0.483  0.035  0.000  0.000  0.014  0.507  0.729 | 1.00  1.14 (1.10, 1.19)  1.22 (1.16, 1.28)  0.85 (0.79, 0.90)  0.55 (0.51, 0.60)  1.36 (1.13, 1.63)  0.90 (0.72, 1.14)  0.90 (0.70, 1.15) | 0.000  0.000  0.000  0.000  0.001  0.387  0.397 |
| **Sex**  Male  Female | 231,495 (72.62)  87,297 (27.38) | 1.00  1.09 (1.07, 1.11) | 0.000 | 1.00  1.03 (1.02, 1.05) | 0.000 |
| **Age** | 318,792 (100) | 0.996 (0.996, 0.996) | 0.000 | 0.998 (0.998, 0.999) | 0.000 |
| **Occupation**  Commercial services/civil servant  Agriculture~  Housekeeping^$^  Education^∆^  Migrant worker  Healthcare  Hospitality  Other | 7,818 (2.45)  249,093 (78.14)  30,802 (9.66)  10,679 (3.35)  2,601 (0.82)  1,009 (0.32)  612 (0.19)  16,178 (5.07) | 1.00  0.42 (0.40, 0.44)  0.78 (0.75, 0.83)  0.68 (0.64, 0.73)  0.46 (0.42, 0.50)  0.84 (0.73, 0.96)  1.03 (0.87, 1.23)  0.60 (0.57, 0.64) | 0.000  0.000  0.000  0.000  0.009  0.696  0.000 | 1.00  0.53 (0.50, 0.56)  0.78 (0.74, 0.83)  0.67 (0.63, 0.72)  0.66 (0.60, 0.73)  0.86 (0.74, 0.99)  1.08 (0.90, 1.30)  0.74 (0.70, 0.79) | 0.000  0.000  0.000  0.000  0.038  0.416  0.000 |
| **Year**  2013  2014  2015  2016  2017  2018 | 56,198 (17.63)  55,815 (17.51)  55,196 (17.31)  49,996 (15.68)  49,843 (15.63)  51,744 (16.23) | 1.00  0.97 (0.95, 0.99)  0.91 (0.89, 0.94)  0.87 (0.85, 0.89)  0.99 (0.97, 1.02)  1.01 (0.99, 1.04) | 0.015  0.000  0.000  0.577  0.232 | 1.00  0.96 (0.94, 0.98)  0.90 (0.87, 0.92)  0.85 (0.83, 0.87)  0.94 (0.92, 0.97)  0.91 (0.88, 0.93) | 0.002  0.000  0.000  0.000  0.000 |
| **Residential Address**  Local  Intra-provincial (within province)  Inter-provincial (between provinces)  Foreign nationality | 310,343 (97.35)  6,215 (1.95)  2,182 (0.68)  52 (0.02) | 1.00  1.28 (1.22, 1.35)  1.78 (1.63, 1.94)  0.41 (0.22, 0.77) | 0.000  0.000  0.006 | 1.00  0.76 (0.72, 0.80)  1.17 (1.07, 1.28)  0.49 (0.25, 0.96) | 0.000  0.001  0.036 |
| **Patient Enrolment Classification**  Consultation due to symptoms  Referral  Contact tracing  Health check  Other | 117,834 (36.96)  103,261 (32.39)  93,183 (29.23)  3,179 (1.00)  1,335 (0.42) | 1.00  2.96 (2.91, 3.01)  6.76 (6.63, 6.89)  1.60 (1.49, 1.73)  8.99 (7.95, 10.16) | 0.000  0.000  0.000  0.000 | 1.00  3.01 (2.95, 3.07)  6.90 (6.76, 7.04)  1.48 (1.37, 1.59)  8.38 (7.40, 9.48) | 0.000  0.000  0.000  0.000 |
| **Diagnosis Institution**  CDC  Hospital  TB dispensary  Other | 278,707 (88.15)  33,104 (10.47)  4,276 (1.35)  69 (0.02) | 1.00  0.79 (0.78, 0.81)  3.72 (3.47, 3.99)  0.94 (0.58, 1.51) | 0.000  0.000  0.784 | 1.00  0.80 (0.77, 0.82)  7.06 (6.56, 7.59)  1.06 (0.63, 1.76) | 0.000  0.000  0.831 |
| **Severely Ill**  No  Yes | 306,534 (96.15)  12,258 (3.85) | 1.00  0.96 (0.93, 0.99) | 0.026 | 1.00  0.84 (0.80, 0.87) | 0.000 |

^~^ Agriculture includes famer, herdsman, fisherman.

^$^ Housekeeping includes housekeeping, childcare, retired and unemployed.

^∆^ Education includes students and teachers.

*Other are represented by: Buyi, Dai, Gelao, Hani, Hui, Jingpo, Kazakh, Kirgiz, Korean, Lahu, Li, Lisu, Manchu, Salar, She, Tibetan, Tu, Uighur, Wa, Yao, Yi, and Zhuang ethnic groups.

**Table S4:** Univariable and multivariable negative binomial regression assessment of factors associated with time from diagnosis to treatment commencement in TB patients registered in Hunan Province, 2013-2018

|  | Univariable coefficient  (95% CI) | Univariable *p* value | Multivariable coefficient  (95% CI) | Multivariable *p* value |
| --- | --- | --- | --- | --- |
| **Ethnicity**  Han  Tujia  Miao  Dong  Yao  Bai  Mongolian  Other* | 0.00  -0.09 (-0.13, -0.05)  -0.35 (-0.40, -0.30)  0.05 (-0.02, 0.13)  -1.22 (-1.31, -1.13)  0.26 (0.05, 0.46)  -0.20 (-0.45, 0.05)  -0.20 (-0.47, 0.07) | 0.000  0.000  0.150  0.000  0.014  0.115  0.141 | 0.00  0.008 (-0.03, 0.05)  -0.24 (-0.29, -0.19)  0.14 (0.07, 0.21)  -1.09 (-1.18, -1.00)  0.35 (0.15, 0.55)  -0.24 (-0.48, -0.003)  -0.30 (-0.57, -0.04) | 0.697  0.000  0.000  0.000  0.000  0.047  0.022 |
| **Sex**  Male  Female | 0.00  0.09 (0.07, 0.11) | 0.000 | 0.00  0.11 (0.09, 0.13) | 0.000 |
| **Age** | 0.002 (0.001, 0.002) | 0.000 |  |  |
| **Occupation**  Commercial services/civil servant  Agriculture~  Housekeeping^$^  Education^∆^  Migrant worker  Healthcare  Hospitality  Other | 0.00  -0.40 (-0.45, -0.35)  -0.11 (-0.16, -0.05)  -0.31 (-0.38, -0.25)  -0.52 (-0.63, -0.42)  -0.12 (-0.27, 0.03)  -0.10 (-0.29, 0.10)  -0.21 (-0.28, -0.15) | 0.000  0.000  0.000  0.000  0.127  0.334  0.000 | 0.00  -0.27 (-0.32, -0.22)  -0.15 (-0.20, -0.09)  -0.25 (-0.31, -0.18)  -0.25 (-0.31, -0.18)  -0.15 (-0.30, 0.001)  0.05 (-0.14, 0.23)  -0.20 (-0.26, -0.14) | 0.000  0.000  0.000  0.000  0.052  0.633  0.000 |
| **Year**  2013  2014  2015  2016  2017  2018 | 0.00  -0.04 (-0.07, -0.02)  -0.03 (-0.06, -0.001)  -0.13 (-0.16, -0.10)  -0.02 (-0.05, 0.01)  0.01 (-0.01, 0.04) | 0.001  0.041  0.000  0.231  0.298 | 0.00  -0.10 (-0.13, -0.08)  -0.002 (-0.03, 0.02)  -0.10 (-0.13, -.008)  0.05 (0.02, 0.07)  0.11 (0.08, 0.14) | 0.000  0.893  0.000  0.001  0.000 |
| **Residential Address**  Local  Intra-provincial (within province)  Inter-provincial (between provinces)  Foreign nationality | 0.00  0.32 (0.26, 0.38)  0.29 (0.20, 0.39)  -0.01 (-0.65, 0.63) | 0.000  0.000  0.975 | 0.00  0.05 (-0.006, 0.11)  0.14 (0.05, 0.24)  0.17 (-0.44, 0.79) | 0.080  0.003  0.574 |
| **Patient Enrolment Classification**  Consultation due to symptoms  Referral  Contact tracing  Health check  Other | 0.00  0.67 (0.65, 0.69)  1.44 (1.43, 1.46)  -0.13 (-0.21, -0.05)  1.44 (1.32, 1.56) | 0.000  0.000  0.002  0.000 | 0.00  0.69 (0.67, 0.71)  1.47 (1.45, 1.49)  -0.07 (-0.15, 0.02)  1.44 (1.31, 1.56) | 0.000  0.000  0.113  0.000 |
| **Diagnosis Institution**  CDC  Hospital  TB dispensary  Other | 0.00  -0.32 (-0.35, -0.30)  -0.08 (-0.15, -0.005)  -0.59 (-1.15, -0.03) | 0.000  0.036  0.038 | 0.00  -0.51 (-0.54, -0.48)  0.47 (0.40, 0.54)  -0.48 (-1.02, 0.05) | 0.000  0.000  0.077 |
| **Severely Ill**  No  Yes | 0.00  -0.13 (-0.18, -0.09) | 0.000 | 0.00  -0.28 (-0.32, -0.24) | 0.000 |

**Table** **S5**: Median time from diagnosis to treatment commencement for **new** TB patients registered in Hunan Province, 2013-2018, by demographic characteristics.

|  | Number of **new** patients (%) | Treatment time (days) **new** patients |
| --- | --- | --- |
| All patients | 305,218 | 1 (IQR 0–15) |
| **Ethnicity**  Han  Tujia  Miao  Dong  Yao  Bai  Mongolian  Other* | 276,961 (90.74)  12,763 (4.18)  8,074 (2.65)  3,827 (1.25)  2,512 (0.82)  458 (0.15)  337 (0.11)  282 (0.09) | 1 (IQR 0–16)  1 (IQR 0–10)  1 (IQR 0–9)  1 (IQR 0–9)  0 (IQR 0–2)  2 (IQR 0–11)  1 (IQR 0–13)  1 (IQR 0–9) |
| **Sex**  Male  Female | 220,703 (72.31)  84,515 (27.69) | 1 (IQR 0–14)  1 (IQR 0–17) |
| **Age**  < 18 years  > 18 years | 7,111 (2.34)  298,107 (97.67) | 2 (IQR 0–17)  1 (IQR 0–15) |
| **Occupation**  Agriculture^~^  Housekeeping^$^  Education^∆^  Commercial services/civil servant  Migrant worker  Healthcare  Hospitality  Other | 237,797 (77.91)  29,469 (9.66)  10,583 (3.47)  7,610 (2.49)  2,511 (0.82)  988 (0.32)  599 (0.20)  15,661 (5.13) | 1 (IQR 0–12)  4 (IQR 0–27)  2 (IQR 0–20)  7 (IQR 0–30)  1 (IQR 0–10)  4 (IQR 0–25)  6 (IQR 0–27)  2 (IQR 0–23) |
| **Year**  2013  2014  2015  2016  2017  2018 | 53,830 (17.64)  53,543 (17.54)  52,989 (17.36)  47,991 (15.72)  47,778 (15.65)  49,087 (16.08) | 1 (IRQ 0–14)  1 (IQR 0–14)  1 (IQR 0–14)  1 (IQR 0–13)  1 (IQR 0–16)  1 (IQR 0–19) |
| **Residential Address**  Local  Intra-provincial (within province)  Inter-provincial (between provinces)  Foreign nationality | 297,023 (97.32)  6,015 (1.97)  2,131 (0.70)  49 (0.02) | 1 (IQR 0–15)  2 (IQR 0–30)  5 (IQR 0–28)  0 (IQR 0–1) |
| **Patient Enrolment Classification**  Consultation due to symptoms  Referral  Contact tracing  Health check  Other | 110,843 (36.32)  99,528 (32.61)  90,476 (29.64)  3,139 (1.03)  1,232 (0.40) | 0 (IQR 0–1)  1 (IQR 0–12)  16 (IQR 0–38)  0 (IQR 0–3)  9 (IQR 2–28) |
| **Diagnosis Institution**  CDC  Hospital  TB dispensary  Other | 267,390 (87.61)  31,116 (10.20)  4,120 (1.35)  68 (0.02) | 1 (IQR 0–15)  0 (IQR 0–9)  11 (IQR 2–18)  1 (IQR 0–6.5) |
| **Severely Ill**  No  Yes | 293,371 (96.12)  11,847 (3.88) | 1 (IQR 0–15)  1 (IQR 0–13) |

^~^ Agriculture includes famer, herdsman, fisherman.

^$^ Housekeeping includes housekeeping, childcare, retired and unemployed.

^∆^ Education includes students and teachers.

*Other are represented by: Buyi, Dai, Gelao, Hani, Hui, Jingpo, Kazakh, Kirgiz, Korean, Lahu, Li, Lisu, Manchu, Salar, She, Tibetan, Tu, Uighur, Wa, Yao, Yi, and Zhuang ethnic groups.

**Table S6:** Univariable and multivariable regression of factors associated with >15-day treatment delay in **new** TB patients registered in Hunan Province, 2013-2018

|  | Number of new patients (%) | Univariable odds ratio (95% CI) | Univariable *p* value | Multivariable odds ratio (95% CI) | Multivariable *p* value |
| --- | --- | --- | --- | --- | --- |
| All patients | 305,218 |  |  |  |  |
| **Ethnicity**  Han  Tujia  Miao  Dong  Yao  Bai  Mongolian  Other* | 276,961 (90.74)  12,763 (4.18)  8,074 (2.65)  3,827 (1.25)  2,512 (0.82)  458 (0.15)  337 (0.11)  282 (0.09) | 1.00  0.74 (0.71, 0.77)  0.61 (0.58, 0.65)  0.79 (0.73, 0.86)  0.20 (0.17, 0.24)  0.74 (0.59, 0.93)  0.86 (0.67, 1.12)  0.70 (0.52, 0.95) | 0.000  0.000  0.000  0.000  0.009  0.266  0.020 | 1.00  0.91 (0.87, 0.96)  0.75 (0.70, 0.80)  0.91 (0.83, 0.99)  0.21 (0.17, 0.24)  0.90 (0.71, 1.15)  0.79 (0.59, 1.05)  0.72 (0.52, 0.999) | 0.000  0.000  0.021  0.000  0.408  0.100  0.049 |
| **Sex**  Male  Female | 220,703 (72.31)  84,515 (27.69) | 1.00  1.11 (1.09, 1.13) | 0.000 | 1.00  1.07 (1.05, 1.10) | 0.000 |
| **Age** | 305,218 | 0.999(0.998, 0.999) | 0.000 | 1.0009 (1.0004, 1.002) | 0.001 |
| **Occupation**  Commercial services/civil servant  Agriculture^~^  Housekeeping^$^  Education^∆^  Migrant worker  Healthcare  Hospitality  Other | 7,610 (2.49)  237,797 (77.91)  29,469 (9.66)  10,583 (3.47)  2,511 (0.82)  988 (0.32)  599 (0.20)  15,661 (5.13) | 1.00  0.45 (0.43, 0.48)  0.85 (0.81, 0.90)  0.64 (0.60, 0.68)  0.41 (0.37, 0.46)  0.81 (0.70, 0.93)  0.88 (0.74, 1.05)  0.68 (0.64, 0.72) | 0.000  0.000  0.000  0.000  0.003  0.156  0.000 | 1.00  0.57 (0.54, 0.61)  0.80 (0.76, 0.85)  0.67 (0.62, 0.72)  0.63 (0.56, 0.71)  0.81 (0.70, 0.95)  0.92 (0.76, 1.11)  0.81 (0.76, 0.87) | 0.000  0.000  0.000  0.000  0.009  0.373  0.000 |
| **Year**  2013  2014  2015  2016  2017  2018 | 53,830 (17.64)  53,543 (17.54)  52,989 (17.36)  47,991 (15.72)  47,778 (15.65)  49,087 (16.08) | 1.00  0.99 (0.97, 1.02)  0.97 (0.94, 0.996)  0.94 (0.91, 0.97)  1.07 (1.04, 1.10)  1.18 (1.15, 1.21) | 0.680  0.026  0.000  0.000  0.000 | 1.00  0.95 (0.92, 0.98)  0.93 (0.90, 0.96)  0.90 (0.87, 0.93)  0.99 (0.96, 1.02)  1.09 (1.05, 1.12) | 0.001  0.000  0.000  0.541  0.000 |
| **Residential Address**  Local  Intra-provincial (within province)  Inter-provincial (between provinces)  Foreign nationality | 297,023 (97.32)  6,015 (1.97)  2,131 (0.70)  49 (0.02) | 1.00  1.73 (1.64, 1.82)  1.74 (1.59, 1.90)  0.70 (0.34, 1.45) | 0.000  0.000  0.341 | 1.00  0.96 (0.91, 1.02)  1.14 (1.03, 1.26)  1.10 (0.50, 2.40) | 0.211  0.010  0.821 |
| **Patient Enrolment Classification**  Consultation due to symptoms  Referral  Contact tracing  Health check  Other | 110,843 (36.32)  99,528 (32.61)  90,476 (29.64)  3,139 (1.03)  1,232 (0.40) | 1.00  3.61 (3.51, 3.72)  14.40 (14.02, 14.79)  1.14 (0.998, 1.31)  8.57 (7.62, 9.64) | 0.000  0.000  0.053  0.000 | 1.00  3.60 (3.50, 3.71)  14.40 (14.01, 14.80)  1.09 (0.95, 1.25)  7.90 (7.01, 8.89) | 0.000  0.000  0.214  0.000 |
| **Diagnosis Institution**  CDC  Hospital  TB dispensary  Other | 267,390 (87.61)  31,116 (10.20)  4,120 (1.35)  68 (0.02) | 1.00  0.75 (0.73, 0.77)  1.38 (1.29, 1.47)  0.64 (0.34, 1.20) | 0.000  0.000  0.165 | 1.00  0.73 (0.70, 0.76)  3.22 (3.00, 3.46)  0.79 (0.40, 1.56) | 0.000  0.000  0.498 |
| **Severely Ill**  No  Yes | 293,371 (96.12)  11,847 (3.88) | 1.00  0.84 (0.81, 0.88) | 0.000 | 1.00  0.71 (0.68, 0.75) | 0.000 |

^~^ Agriculture includes famer, herdsman, fisherman.

^$^ Housekeeping includes housekeeping, childcare, retired and unemployed.

^∆^ Education includes students and teachers.

*Other are represented by: Buyi, Dai, Gelao, Hani, Hui, Jingpo, Kazakh, Kirgiz, Korean, Lahu, Li, Lisu, Manchu, Salar, She, Tibetan, Tu, Uighur, Wa, Yao, Yi, and Zhuang ethnic groups.

**Additional file**

Fig S1: Median time to diagnosis for TB patients registered in Hunan Province 2013-2018.

Table S1: Sensitivity Analysis: Univariable and multivariable regression of factors associated with 14-day diagnosis delay in TB patients registered in Hunan Province, 2013-2018

Table S2: Univariable and multivariable negative binomial regression assessment of factors associated with time to diagnosis in TB patients registered in Hunan Province, 2013-2018

Table S3: Sensitivity Analysis: Univariable and multivariable regression assessment of factors associated with 1-day treatment delay in TB patients registered in Hunan Province, 2013-2018

Table S4: Univariable and multivariable negative binomial regression assessment of factors associated with time from diagnosis to treatment commencement in TB patients registered in Hunan Province, 2013-2018

Table S5: Median time from diagnosis to treatment commencement for **new** TB patients registered in Hunan Province, 2013-2018, by demographic characteristics.

Table S6: Univariable and multivariable regression of factors associated with >15-day treatment delay in **new** TB patients registered in Hunan Province, 2013-2018
